# Clinical Trials and Administration of Zika Virus Vaccine in Pregnant Women: Lessons (that Should Have Been) Learned from Excluding Immunization with the Ebola Vaccine during Pregnancy and Lactation

**DOI:** 10.3390/vaccines6040081

**Published:** 2018-12-04

**Authors:** David A. Schwartz

**Affiliations:** Medical College of Georgia, Augusta University, Augusta, GA 30912, USA; davidalanschwartz@gmail.com

**Keywords:** Zika virus, vaccine, Zika vaccine, congenital Zika syndrome, pregnancy, Ebola virus, Ebola vaccine, women’s rights, clinical trials, epidemic, vaccination, congenital infections, exclusion pregnant women

## Abstract

As evidenced from recent epidemics, both Ebola and Zika virus infection are potentially catastrophic when occurring in pregnant women. Ebola virus causes extremely high rates of mortality in both mothers and infants; Zika virus is a TORCH infection that produces a congenital malformation syndrome and pediatric neurodevelopmental abnormalities. Production of efficacious vaccines has been a public health priority for both infections. Unfortunately, during the clinical trials and subsequent deployment of a vaccine for the Ebola virus, pregnant and lactating women were, and continue to be, excluded from receiving the life-saving vaccine. The most serious consequence of Zika virus infection, congenital Zika syndrome, results from fetal infection during pregnancy. Thus, pregnant women have a major stake in the ongoing development of a vaccine for Zika virus. The exclusion of pregnant women from the development, clinical trials and administration of a potential Zika vaccine unfairly deprives them and their infants of the protection they need against this potentially catastrophic intrauterine infection. When creating policy about these issues, it is important to critically evaluate vaccine safety in pregnancy in the context of the substantial risk of infection for the pregnant woman and her fetus in the absence of immunization.

## 1. Introduction

The Zika virus outbreak was first recognized in Northeastern Brazil in early 2015, spread rapidly through South American countries, into Central America and the Caribbean, became a global pandemic, and now occurs as an endemic arboviral infection throughout much of the Western hemisphere [1]. Spread of the virus is not just limited to the natural ecology of its major insect vector, the mosquito *Aedes aegypti*, because sexual transmission of this flavivirus has been found to occur from males to their female partners [2]. The Zika virus is asymptomatic in the majority of infected persons, with only 1 in 5 persons exhibiting symptoms. Although Guillain-Barré syndrome has been strongly associated with Zika virus infection [3], its occurrence is rare, and for the overwhelming majority of non-pregnant adults, the infection is of little medical consequence beyond a transient febrile illness accompanied by rash, conjunctivitis, muscle and joint pains, and headache.

The most significant complications of Zika virus occur when pregnant women become infected, as the Zika virus has now been recognized as a TORCH infection [4]. TORCH is an acronym for a group of vertically transmitted infections occurring in pregnancy, and which originally consisted of Toxoplasmosis, Other, Rubella, Cytomegalovirus and Herpes virus, but now contains additional infectious agents [4]. Vertical transmission of the Zika virus can infect the fetus and cause a unique pattern of birth defects termed the congenital Zika syndrome (CZS) [5,6]. This syndrome encompasses the spectrum of abnormalities resulting from intrauterine fetal infection resulting from transplacental passage of the virus, and includes changes resulting from fetal brain disruption sequence including microcephaly, collapse of the skull and a pattern of destructive changes to the brain, ocular pathology including macular scarring and focal pigmentary retinal mottling, joint disease including congenital contractures, prominent early onset hypertonia with symptoms of extrapyramidal involvement, and additional abnormalities [7]. A study by the U.S. Centers for Disease Control and Prevention (CDC) has shown that up to 1 in 7 of infants born to mothers with Zika infection during pregnancy have a birth defect, neurodevelopmental abnormality, or both [8]. In addition to having malformations from congenital Zika syndrome, it has been found that as infants with CZS become older, there is an increasing incidence of such neurodevelopmental problems as epilepsy, vision loss, and developmental delays [9]. As a result, of the significant risks of having a child with congenital Zika syndrome or other abnormality, pregnant women globally have concerns about Zika virus infection.

Although the worldwide prevalence of Zika infection has dramatically decreased [10], the infection remains endemic in many of the affected countries and has the potential to once again become epidemic in other parts of the world. A high priority in public health is the development of a safe, efficacious and affordable vaccine for use in pregnant women in order to prevent the terrible consequences of intrauterine fetal infection. A wide range of vaccine candidates are currently under development, including traditional inactivated and live vaccines as well as DNA, mRNA, and protein subunit vaccines. These experimental vaccines are in varying stages of preclinical and clinical testing, with some having reached Phase I and Phase II trials. 

There is little doubt that the development of an effective Zika vaccine will have its greatest benefit in preventing pregnant women from acquiring the infection and transmitting the virus to their unborn infants. However, this population has historically been excluded from clinical testing of experimental vaccines—potential harm to the fetus, physiological complexity of pregnancy, and the threat of punitive legal actions have all been cited as some of the reasons contributing to the exclusion of pregnant women from clinical vaccine trials, as well as receiving them following their approval for use. Most recently, the exclusion of pregnant women from both experimental vaccine clinical trials as well as administration of highly effective vaccines during epidemics has been exemplified with a series of Ebola virus outbreaks in Africa. 

There is little in common biologically between the Zika and Ebola viruses. The Ebola virus is a filovirus, has a probable reservoir in bats, and its major mechanism of transmission is person-to-person through infected body fluids; the Zika virus is a flavivirus with primates as the main reservoir, and is an arbovirus whose major transmission mechanism is through the bite of infected *Aedes* sp. mosquitoes. Maternal infections from Ebola virus produce severe illness and, more often than not, maternal death; maternal infections from the Zika virus typically produce no symptoms in the mother or, at most, a mild illness. However, both viruses cause maternal viremia, and can be transmitted from an infected mother to her fetus through the placenta. When this occurs with Ebola virus, in early pregnancy there can be a spontaneous abortion, or later in gestation, the fetus either dies in utero or shortly following birth; there is no malformation syndrome—the maternal-fetal transmission rate is essentially 100%. In contrast, maternal Zika infection may produce a syndrome of fetal malformations including microcephaly or neurodevelopment abnormalities in approximately 1 in 7 fetuses [8]. What these two viral infections do have in common is that (1) they have both recently produced global pandemics that have disproportionately affected pregnant women and their infants; (2) both viruses can infect fetuses transplacentally and produce differing, but devastating, fetal and neonatal injuries; (3) an effective vaccine has recently been produced and tested for the Ebola virus; and (4) vaccines are in development for the prevention of maternal Zika virus infection and congenital disease. However, the clinical testing and subsequent distribution of the Ebola vaccine has excluded pregnant and lactating women and their infants and continues to do so despite continuing outbreaks. Will this restriction be repeated during the clinical trials and administration of Zika vaccine candidates? As this communication will discuss, excluding pregnant women from vaccine trials and distribution is an unfortunate decision, as both the Ebola and Zika virus can cause tragic injuries to the fetus, and it is hoped that women will be included throughout the development and testing of Zika virus vaccines.

## 2. Pregnant Women, Infants and Vaccines

Throughout the world, and especially in resource-poor countries, infectious diseases continue to be a major and preventable cause of mortality and morbidity in children during the first four years of life—at least 1.5 million deaths of children less than 5 years of age occur annually from vaccine-preventable diseases [11]. Infants do not develop protective immunity in response to many vaccines administered after birth, and many vaccines are not administered until infants are at least several months of age, leaving an interval where they remain vulnerable to infection [12]. Pregnant women may also succumb to infections, sometimes in high numbers, as has been seen during outbreaks of Ebola virus disease, Lassa fever, and others [13,14]. Vaccine-preventable infections are a leading cause of morbidity in pregnant women [12]. As a result, immunization of pregnant women has emerged as a potentially efficacious public health strategy to prevent not only morbidity and mortality in the mother, but also the fetus and newborn [15]. Maternal vaccination not only protects the mother from the effects of infectious diseases during her pregnancy, but it permits vaccine-induced maternal antibodies to be transferred transplacentally to the fetus, as well as to the newborn and infant in colostrum and via breast milk for protection against diseases before routine childhood immunization is begun [15]. 

From a historical perspective, the needs of pregnant women have never been prioritized in the either the development or clinical testing of pharmaceuticals or vaccines. Even when new biomedical products reach the marketplace, information on their safe use for pregnant women and the fetus is usually incomplete, or even lacking. All vaccine formulations that are currently in use in pregnant women were, in fact, initially developed and tested for in non-pregnant persons [12]. Pregnancy has always been problematic for the testing and administration of both drugs and vaccines. Considered as a hazardous period for a variety of reasons, it can be difficult to assure the safety to the mother and developing embryo and fetus of drugs and vaccines, and thus caution has traditionally guided their administration during pregnancy. The possibility of side effects occurring in the mother may have a deleterious effect on progress of the pregnancy, labor and delivery; effects on the placenta and its function may be uncertain, transplacental passage of the drug may result in teratogenic, developmental and toxic effects to the fetus; intrauterine fetal demise may occur; and there is the danger of passage of the drug to the newborn infant following delivery through breastmilk [16]. The variables of dosage, timing, form and method for administration of vaccines during pregnancy have also been problematic as compared with non-pregnant recipients. In addition, because pregnancy is a complex immunological state, it can affect the manner in which a drug or vaccine interacts with the host—alterations in the maternal immune response, believed to permit the mother to tolerate a semi-allogeneic fetus, can potentially interfere with the specific immune response to pathogens and alter susceptibility of the maternal-fetal pair to infections [12,17]. Even in the non-pregnant state, the hormonal, genetic, immunological and environmental differences between females and males can affect their immune responses and the sex-related outcome of vaccination.

The potential risks for drug administration during pregnancy were perhaps best illustrated during the 1950s and early 1960s, when a syndrome of congenital limb malformations, termed phocomelia, developed in many thousands of infants from mothers who were given the drug thalidomide as a treatment for morning sickness during pregnancy [18]. 

In contrast, no maternal vaccine has been proven to result in birth defects. The use of inactivated vaccines and toxoids is usually considered safe during pregnancy. Maternal immunization is highly recommended against the influenza virus, which can produce morbidity and even be life-threatening to both mother and fetus when occurring during pregnancy [19,20]. The safety of their use in pregnancy, and efficacy in preventing both maternal and fetal complications illness from influenza, have been repeatedly shown both during seasonal outbreaks as well as pandemics of the infection [20]. Newer formulations of the influenza vaccine utilize an oil-in-water adjuvant; recent studies of the safety of these vaccines have not indicated any significant effects on pregnancy outcomes [20,21], although these data are-based mostly upon the use of monovalent H1N1 vaccines. 

Meningococcal vaccines have been widely distributed in the “meningitis belt” in sub-Saharan Africa, including immunization of pregnant women. Studies of conjugated, bivalent and tetravalent polysaccharide meningococcal vaccines have revealed no adverse maternal or infant outcomes, although the data are limited [20,22]. 

The prevention of neonatal tetanus by administration of a tetanus toxoid vaccine to pregnant women is a well-established public health practice, especially in resource-poor countries. In a retrospective study of the VAERS (Vaccine Adverse Event Reporting System) database for 2005–2010, Zheteyeva et al. [23] did not identify any adverse maternal, fetal and infant outcomes following vaccination with a reduced amount of diphtheria toxoid, tetanus toxoid, and acellular pertussis vaccine (dTap).

The use of live attenuated virus vaccines may be more problematic when considering their use in pregnant women, as theoretically a live attenuated virus could be transmitted across the placenta to the fetus. Although live attenuated virus vaccines have generally not been recommended for use during pregnancy, their inadvertent administration to pregnant women during mass vaccination campaigns has been documented [20]. In those cases where there has been clinical follow-up available following unintentional use of live virus vaccines in women who were pregnant, only rarely has there been associated harm to the fetus [24]. 

Prevention of congenital rubella syndrome has utilized an attenuated live virus vaccine since the 1960s. It is typically given in combination with vaccines for mumps and measles, the MMR vaccine. Extensive clinical follow-up and the results of multiple national registries has indicated that although attenuated rubella and mumps viruses can cross the placenta and infect the fetus, there is no increased risk for spontaneous abortion fetal malformation [20,25].

The polio vaccine uses an attenuated virus and, similar to the rubella virus vaccine, has been used in mass vaccinations since the 1960s. There is no evidence that polio virus vaccine administered to pregnant women has any adverse fetal or neonatal outcome [20].

Yellow fever vaccine came into use in 1938—this attenuated 17D strain was initially developed by Max Theiler and Hugh Smith at the Rockefeller Institute. More than 600 million doses of the vaccine have been distributed, and the effects on several hundred pregnant women and their infants have been studied. The risks of yellow fever vaccination to the mother and fetus appear to be similar to that for the general population [20]. However, yellow fever vaccination is not currently recommended for pregnant women, except under circumstances of an epidemic or travel to an endemic area [26].

It was the emergence of the acquired immunodeficiency syndrome (AIDS) pandemic in the 1980s, together with the recognition of vertical transmission of the human immunodeficiency virus (HIV) from mother to infant, that helped provide investigators impetus to enroll pregnant women in the early phases of anti-retroviral drug trials—even prior to the completion of experimental animal studies. This was because the life-threatening nature of AIDS was believed to justify an unknown risk to the fetus in order to potentially extend the life of the mother [27].

The conservative attitude restricting, and in many cases banning, the enrollment of pregnant women in drug and vaccine trials has persisted up to the present times. Even the participation of non-pregnant women in experimental trials remained restricted until 1993, when the policy was changed so that both sexes were recruited into drug studies in order to determine gender-based differences [28]. This policy shift did not include pregnant women—less than 20 years ago, the United States Food and Drug Administration (FDA) still had a policy of excluding from experimental drug trials those women “of childbearing potential” [29]. More recently, women are being included in drug trials for non-obstetric conditions under the condition that they are not pregnant, and that they do not intend to become pregnant, including the use of birth control [30]. Perhaps the clearest and most permissive clearance for the participation of pregnant women in experimental studies originates with the Joint United Nations Programme on HIV/AIDS/World Health Organization (UNAIDS/WHO) ethical guidance for HIV prevention trials [31]. Guidance Point 9 from this document states, “*Researchers and trial sponsors should include women in clinical trials in order to verify safety and efficacy from their standpoint, including immunogenicity in the case of vaccine trials, since women throughout the life span, including those who are sexually active and may become pregnant, be pregnant or be breastfeeding, should be recipients of future safe and effective biomedical HIV prevention interventions. During such research, women’s autonomy should be respected and they should receive adequate information to make informed choices about risks to themselves, as well as to their fetus or breast-fed infant, where applicable*”.

Unfortunately, despite this sentiment, pregnant women are usually still excluded from experimental trial of drugs and vaccines that do not target obstetric conditions. This policy engenders serious bioethical questions. According to Anne Lyerly, MD, MA, Professor of Social Medicine and the Associate Director of the University of North Carolina Center for Bioethics [32], “*People tend to think first about the ethical problems of including pregnant women in research”. “In this case, the gravest ethical problem would be if we failed to include them, since it is pregnant women—and their babies—who will face the most serious consequences of infection*”.

## 3. Development and Clinical Testing of the Ebola Vaccine during the 2013–2015 West Africa Epidemic

As was evidenced during the global Zika virus pandemic, pregnant women, their fetuses and infants are generally the most vulnerable members of society during an infectious disease outbreak [33,34]. The Ebola virus, a filovirus agent of hemorrhagic fever that is unrelated to Zika virus and is not arthropod borne, also has its greatest deleterious effect on pregnant women and infants. Similar to the Zika virus, Ebola virus was first discovered in Africa. It was initially recognized in 1976 in what was then called Zaire (now the Democratic Republic of Congo, or DR Congo), where it resulted in a limited outbreak in the rural town of Yambuku that infected 316 people and caused 280 deaths over a period of eleven weeks [35]. During this outbreak, there were 73 deaths among the 82 pregnant women infected with Ebola virus, a case fatality rate (CFR) of 89% [36]. Since then, there have been many more outbreaks of the Ebola virus, and despite the limited data available on clinical outcomes of infected pregnant women and their infants from these past epidemics, in those incidents where these figures were available, the outlook for their survival has not been optimistic. For example, during the Kikwit, Zaire epidemic of Ebola virus that occurred in 1995, 14 of 15 pregnant women with Ebola virus disease (EVD) died—a CFR of 93% [37]. No fetuses were reported to have survived the infection [38]. During the outbreaks of EVD that occurred prior to the 2013–2015 West Africa epidemic, there were no effective antiviral therapeutic agents or vaccines available—treatment was limited to supportive care.

The West African Ebola Epidemic that began in late 2013 and rapidly spread through Guinea, Sierra Leone and Liberia was catastrophic, affecting 28,616 persons and causing 11,310 recorded deaths by the time it ended in 2016—the true number of cases and deaths is likely greater [14]. Based upon the previous EVD outbreaks, the prognosis was so poor for pregnant women and their fetuses that at the start of the West African Ebola epidemic that it was predicted that greater than 90 percent of infected pregnant women and 100 percent of fetuses would die as a result of EVD. One interview conducted early in the outbreak reported that a representative from a non-governmental organization (NGO) had opined that the survival rate for expectant mothers was virtually zero [39]. In a report published in 2015 [40], the probability for maternal and infant survival of EVD was summarized as follows, “*Present data suggests that maternal mortality remains high (approximately 95%) and peri-natal mortality virtually 100% for infected pregnant women*”.

During the West African epidemic, specific antiviral compounds and experimental vaccines became available for limited experimental testing or, in some cases, for compassionate use. The trials were organized through a committee—the World Health Organization Research Ethics Review Committee, or WHO-ERC—that evaluated and approved proposed studies. They critically examined 24 new and 22 amended protocols for experimental studies that included both interventional (drug, vaccine) and observational studies [41]. One of the most problematic decisions faced by the committee members in the design, approval and implementation of these protocols was the inclusion of pregnant women and children [41]. It was recognized that exclusion of pregnant women and children from drug and vaccine trials undermined ethical principles of justice—fairness, equity and maximization of benefit. In addition, exclusion of these groups from clinical trials would deny them the potential life-saving benefits from an infection with a high mortality rate, at the time believed to be 100% in fetuses and neonates and approximately 90% in pregnant women [14,42]. These survival data were of immediate relevance in considering the risk/benefit relationship in enrolling pregnant women and infants in clinical trials, as addressed in by the WHO Ethics Working Group meeting on 20–21 October 2014 [43], “*It is ethically important to ensure that vulnerable populations such as pregnant women and those with diminished autonomy such as children or those with mental incapacities are not arbitrarily excluded from trials. Instead their inclusion into clinical trials should be guided by a risk benefit analysis and the ability to secure adequate consent*”.

Because the clinical outcomes data from prior Ebola virus epidemics demonstrated extremely high case fatality rates for pregnant women, as well as the almost certain deaths of their fetuses and newborns, the WHO committee decided to support the inclusion of pregnant women together with their fetuses in the planned clinical trials of both drugs and vaccines. This decision was also based upon their belief that pregnant mothers had a greater interest in, as well as a right to decide, the fate of themselves and their unborn children than did administrators, sponsors, investigators or committee members, and thus pregnant women should be granted the same rights for decision-making as non-pregnant women [37]. 

By the closing days of the West African Ebola epidemic, the WHO-ERC had reviewed fourteen interventional trial protocols and two MEURI proposals [41]. All of the protocols for vaccine clinical trials excluded pregnant women. The proposed clinical trials for two promising antiviral drugs—brincidofovir and favipiravir—also excluded women who were pregnant for a variety of reasons. Despite efforts by the WHO-ERC as well as the Médecins Sans Frontières (MSF) Ethics Review Board and Inserm Institutional Review Board to the applicants to reconsider the exclusion of pregnant women, the necessity for rapid implementation of the trials in the field took priority over the delays that would have been encountered in pursuing revision of the protocols to include pregnant women [37,41]. This exclusion even continued when the clinical trial of the experimental live attenuated vaccine rVSVΔG/ ZEBOV-GP (Merck) demonstrated protective effects in non-pregnant adults, and the WHO-ERC and Data Safety Monitoring Board requested that pregnant women receive the vaccine—42 pregnant women were denied participation in the trial [37,44]. In addition, there were yet some pregnant women who were unintentionally and unknowingly included in the vaccine trials—because pregnancy tests were not routinely performed, and pregnant women were identified on the basis of self-reporting, greater than 20 pregnant women were administered the vaccine [37,44]. According to Dr. Severine Caluwaerts, an MSF obstetrician who had a conversation with Dr. Ana Maria Henao-Restrepo of the WHO, these 20 women have apparently suffered no ill-effects [30,45], “*She* [Dr. Henao-Restrepo] *hasn’t published her data yet on the 20 women who were accidentally vaccinated*”, “*She just said orally at a conference that they are fine and that the babies are fine, but we have not seen it in writing. MSF would love to be able to see this in writing, also to be able to advocate more for the vaccine for pregnant women*”.

Enrollment into clinical trials and access to potentially life-saving drugs and vaccines for pregnant women was difficult despite the obvious risk/benefit considerations; in some cases, the pharmaceutical corporations that produced the products would simply not permit their administration to women who were pregnant, or the insurers would not provide insurance to pregnant women [46]. From the start to the end of the West African Ebola epidemic, pregnant women, together with their fetuses and newborns, had been systematically excluded from essentially all drug and vaccine clinical trials. Eventually, some pregnant women received access to favipiravir, but only following extensive negotiations between the manufacturer and Médecins Sans Frontières. The sole surviving newborn with EVD, Baby Nubia, had received experimental treatments from Médecins Sans Frontières including ZMapp and the broad-spectrum antiviral GS-5734 outside of the clinical trial protocol [46,47]; her mother had been denied access to potentially protective vaccination due to her pregnant condition and died of Ebola infection shortly after delivery [38]. 

Following several isolated flare-ups of infection in the three involved countries, West Africa was declared Ebola-free in June 2016. No fetus had survived a maternal infection; Baby Nubia was the only newborn reported to have survived. Although the exact maternal mortality rate will likely never be known, it has been estimated to be at least 80 percent during this epidemic, was likely higher in some regions, and perhaps approached the 90 percent or greater fatality rate seen among pregnant women in previous Ebola outbreaks. Those pregnant women who survived Ebola infection had a miscarriage rate of essentially 100 percent [46,48,49]. 

## 4. Continued Exclusion of Pregnant Women from Receiving Ebola Vaccine during the 2018 Epidemics in Democratic Republic of Congo

In 2018, a new outbreak of Ebola virus occurred in the Équateur province of northwestern Democratic Republic of Congo—it was the 9th outbreak of Ebola to occur in that country [50]. On 8 May, officials reported that 17 persons had died from Ebola virus infection near Bikoro, a small market town lying on Lake Tumba south of Mbdanka, near the neighboring Republic of the Congo. The initial case was a police officer, and following his funeral, 11 members of his family developed the infection, of whom 7 who had cared for him or attended his funeral died. As the numbers of cases increased, on 17 May, the first case was reported from Mbadanka, the capital city of Équateur province and a bustling port city of over one million persons located on the Congo River. It was the first time that Ebola virus had entered a city in the DRC, and reawakened fears of what had occurred when the infection reached urban areas during the West African epidemic. There was also concern that the virus could spread via river traffic to the capital city of Kinshasa, a city of approximately 11 million, as well as to Brazzaville, the capital city of the Republic of the Congo, both of which lie on the Congo River. The World Health Organization feared that the outbreak could spread across national borders to nine other countries as well, and Ebola virus deaths were being reported among health care workers, evoking recent memories of the West African epidemic. Fortunately, during this outbreak, the recently developed Merck live-attenuated vaccine to Ebola virus, recombinant vesicular stomatitis virus-Zaire Ebola virus or rVSV-ZEBOV, was available. This live-attenuated vaccine had previously been tested during the West African epidemic. Ring fence vaccinations were rapidly implemented across the affected areas—using this method, contacts of those infected, followed by contacts of those contacts, were vaccinated, as were health care workers, laboratory personnel, surveillance workers and people involved with burials. Unfortunately, the previous policies of exclusion of pregnant and lactating women were once again implemented, excluding these women from receiving the potentially life-saving vaccine. The continuance of this policy following the West African epidemic, when pregnant women and children had not been permitted to receive experimental antiviral drugs or vaccines, was considered to be indefensible by some in the public health community [48,49,51], especially since there had never been a mother-infant pair that survived Ebola infection. By the close of the epidemic on 24 July 2018 there were 54 confirmed or suspected cases and 33 deaths with an overall CFR of 61%.

Only one week following the cessation of the Équateur province outbreak, Ebola returned to a different region of the DRC [52,53]. A woman from Mangina, a town in North Kivu district in the northeastern part of the country, had been seen at a local health center on 19 July 2018 for a cardiac condition, and died at home with symptoms of hemorrhagic fever on 25 July following her discharge. Several members of her family subsequently developed the same symptoms, dying soon afterwards [52]. An investigation revealed an additional six cases, and following confirmation of the disease as Ebola virus, an outbreak was declared on 1 August. The area of this epidemic was especially challenging from the standpoint of epidemiological surveillance, medical intervention and control. North Kivu is densely populated, borders Uganda to the east and Rwanda to the south and is an active conflict zone. The Kivu conflict had been ongoing since 2004, with more than 100 armed groups operating in this region [52]. Violence and crime are common and there are intensive military operations ongoing—the administrative center of the district, Beni, is under military rule. The so-called “red zones” are inaccessible to public health workers due to fighting and the risk of kidnapping. Vaccination was begun on August 8th, and a ring vaccination program was implemented to try to halt spread of the virus. In this program—similar to that used in the Équateur province Ebola outbreak—the rVSV-ZEBOV vaccine was offered to contacts of known cases and the contacts of contacts, including any individual over 1 year of age—except pregnant and lactating women. The Ministry of Health of the DRC, together with the WHO and other partners, made the decision that pregnant and lactating women would, once again, be excluded from receiving the Merck vaccine rVSV-ZEBOV, the only Ebola vaccine to have completed efficacy testing [48,49]. Three public health experts from Johns Hopkins University [49] wrote, “*The rVSV-ZEBOV vaccine will give pregnant women, and the children they are carrying, a chance to live. Without it, most of the pregnant women infected with Ebola, and almost all of their infants, will die*”.

According to Ruth Karron, MD of the Johns Hopkins University Bloomberg School of Public Health [48], “*People will say, ‘Well, where’s the evidence this vaccine is safe in pregnancy?’ Well, how can we know if we’ve never tested it in pregnancy? ‘Well, we couldn’t test it in pregnancy because it is not safe to test it in pregnancy’” “And if you can’t get off that … little spinning wheel, pregnant women and their babies are going to be left out indefinitely*”.

As the Ebola infection spread, the district of Ndindi in Beni city became the major focus of the epidemic. WHO officials have commented that responders were reporting a higher-than-expected number of illnesses in women, accounting for 58% of affected persons. On 4 September the city of Butembo, with a population of almost one million people, reported its first fatality in the Ebola outbreak. As of 30 November 2018, there were 434 cases of Ebola infection (386 confirmed and 48 probable), including 252 deaths (204 confirmed and 48 probable) in fourteen health zones in North Kivu Province, as well as three health zones in Ituri Province [54]. This is now the second largest epidemic of Ebola virus in history, surpassing the Uganda epidemic that occurred in 2000. Since the start of vaccination on August 8th, there have been 38,353 persons vaccinated. Among the new cases that occurred in the Kalunguta health zone of North Kivu, a 6-day old neonate died of Ebola virus disease on November 4th. The infant’s mother had developed symptoms of Ebola infection 5 days before delivering her son; neither had received the vaccine. Also among the newest reported cases have been 7 newborn babies and infants ages less than 2 years, 3 children aged 2-17 years, and 3 mothers who were pregnant or breastfeeding. Challenges in the control of this outbreak are similar to those existing during the 2013–2015 West African epidemic—families concealing persons with potential or probable infection, refusals to permit health care providers to take patients to the Ebola treatment center (ETC) or to be quarantined, delays by persons in reaching the ETC after developing symptoms, refusal of treatment or, in this present outbreak, vaccination, unsafe burials, weak infection prevention and control procedures in health facilities leading to disease transmission, and the occurrence of violent incidents against medical staff and care facilities. As in previous Ebola outbreaks, a significant number of health care workers have become infected and died. Dr. Séverine Caluwaerts, an obstetrician gynecologist and expert on Ebola virus disease in pregnancy who was instrumental in defining the treatment of pregnant women during the West African Ebola virus outbreak, stated [48], “*We absolutely don’t agree with the decision of WHO and the Congolese government not to administer the vaccine to pregnant women” “We would at least give them the choice” “The risk-benefit is so much in favor of giving the vaccine*”.

Dr. Peter Salama, an epidemiologist and Deputy-Director General of Emergency Preparedness and Response for the WHO, said that the final decision on whom to vaccinate rests with the DRC government, not the WHO. Dr. Salama said [48], “*I’m not trying to absolve WHO of responsibility here” “But ultimately it is up to the country’s research and ethical review bodies to weigh up what is simply not a black-and-white decision*”.

As of 10 October, 90 vaccination rings had been defined, in addition to 31 rings of health and frontline workers. The Ebola vaccine had been administered to 3439 children over the age of one year—no pregnant or lactating women received the vaccine. Unfortunately, the ban on providing the Merck Ebola vaccine to pregnant and lactating women has not been lifted. Brittany Kmush, PhD, a vaccines expert at Syracuse University, addressed their exclusion [51], “*Most clinical trials automatically exclude pregnant women on the grounds of unknown risks for the fetus, often without biological justification. However, most ethics experts now agree that, in life-threatening situations, pregnant women should be included in clinical trials. This certainly applies in the case of Ebola virus, which has about 90 percent maternal mortality and near 100 percent fetal and neonatal mortality in infected pregnant women”. “The research community needs to recognize the autonomy of pregnant women and let them decide for themselves if they wish to participate in research*”.

One of the oft-cited reasons that the Merck vaccine had been withheld from the ring vaccination efforts during the two outbreaks in DR Congo is the uncertainty surrounding using a live virus vaccine in pregnant women. However, the rVSV-ZEBOV vaccine utilizes a recombinant form of the vesicular stomatitis virus (VZV), which is a rhabdovirus infecting cattle, swine and horses—it is essentially harmless to humans, producing only a flu-like illness [45]. 

## 5. Pregnant Women, Clinical Testing and Administration of the Zika Vaccine

Following the genetic sequencing of the Zika virus genome and the cloning of Zika genes from Brazilian viral isolates in mid-2015, development of Zika vaccines was initiated. The WHO and UNICEF published an updated Target Product Profile for a Zika vaccine in 2017—this presented both the minimal, as well as the preferred, product characteristics of a Zika virus vaccine that would target the prevention of fetal infection and congenital Zika syndrome in an emergency scenario [55]. According to this report, a Zika vaccine should be used to prevent clinical illness in individuals aged 9 years and over in 80% of the population (or minimum of 70%) based on the assumption that a reduction in viremia to a particular level would be associated with prevention of clinical illness and infection of the fetus for at least one year following administration of the primary vaccine series.

Many different approaches and technologies are being investigated in the development of Zika vaccines—these include inactivated Zika virus, attenuated Zika virus strains, live or inactivated viral recombinants that express Zika virus proteins (including adenovirus, lentivirus, dengue virus, modified vaccinia virus Ankara, and the measles virus), viral-like particles expressing Zika virus membrane proteins, DNA plasmid vaccines, mRNA-based vaccines, recombinant protein vaccines, peptide-based vaccines and protein-nanoparticle conjugates [56]. There have been more than 45 vaccine candidates identified in the discovery phase, with over 25 in nonclinical development, and 9 currently in the clinical phase of testing [57]. There are four types of candidate vaccines for Zika virus that have progressed into the Phase 1 level of clinical trials involving of human testing—three vaccine candidates are DNA, one is modified RNA, four are purified formalin inactivated virus, and one is live measles vectored [38]. Preliminary results have indicated that an immunogenic vaccine to the Zika virus can be developed; however, it remains problematic whether a clinically efficacious vaccine can be prepared. Because of the threat of congenital Zika virus infection, it is of paramount importance that a Zika vaccine prevent viral infection of the female reproductive tissues, as the Zika virus has been identified and localized within cells not only in the decidual (endometrial) tissues of the mothers’ uterus, but also in the placenta [58,59,60,61].

In considering the inclusion of pregnant women in the testing of candidate vaccines for Zika virus infection, the potential risk to the fetus of acquiring congenital Zika syndrome in a nonimmunized mother must be taken into account. Pregnant women will most often not be aware that they have become infected; there is no treatment for this TORCH infection for either mother or fetus, and the severe nature of the fetal central nervous system injuries and associated malformations is not only devastating to the infant, but to the mother and family as well. The dreadful consequences of congenital Zika syndrome involve not only the affected children but also their parents, families and their communities. For those women who give birth to infants with congenital Zika syndrome they can begin a lifetime of suffering together with their children. They experience significant and on-going emotional and psychological distress, and often bear much if not most of the burden of caring for their children’s intensive needs. In many cases, these families are from the lower socioeconomic strata of their countries, and they have little or no monetary resources to provide adequate care for a child with severe neurological injuries. Mothers of children with CZS are often stigmatized and, in some cases, abandoned by their partners and communities [62]. The threat of having a child with Zika infection has resulted in many women seeking abortions in countries where abortion is criminalized and the penalties to the mother for having one can be most severe in terms of criminal prosecution—this can also lead to seeking of unsafe abortions, producing maternal morbidity and mortality [63]. Vaccine expert Michael S. Diamond, MD, PhD, the Herbert S. Gasser Professor of Medicine at Washington University School of Medicine in St. Louis, has stated [64], “*In general, most doctors don’t want to vaccinate during pregnancy on the outside chance that the immune response itself could harm the fetus”, “But if you’re in an area where Zika is circulating, you might vaccinate during pregnancy because the risk of Zika infection is worse than some theoretical risk of immune-mediated damage*”.

In addition to developing a prophylactic Zika vaccine that prevents the population (including pregnant women) from developing infection following the bite of an infected mosquito, a therapeutic vaccine would be especially important for use in pregnant women. Unlike preventive vaccines, therapeutic vaccines are used for treatment. In the case of a therapeutic vaccine, a woman who develops Zika infection shortly before, or even during, pregnancy would be vaccinated to help prevent infection of the fetus and the development of the congenital syndrome. There are studies of mouse and human monoclonal antibodies to Zika virus that have been conducted both in mice and non-human primates that hold promise for the development of such a vaccine [65,66]. Magnani et al. [66] administered a cocktail of three anti-Zika monoclonal antibodies to rhesus macaques one day prior to a Zika virus challenge—the macaques did not develop any detectable viremia. Sapparapu et al. [65] have used monoclonal antibodies to prevent maternal-fetal viral transmission in a murine model.

In designing an effective vaccine against Zika virus for use in pregnant women, antibody-dependent enhancement (ADE) of infection must be considered. Flaviviruses such as Zika virus, dengue virus, West Nile virus, yellow fever virus and others exhibit antigenic cross-reactivity, and in many cases exhibit a similar geographic distribution. As a result, preexisting poorly neutralizing antibodies to the shared immunogenic epitopes that these viruses possess can result in exacerbation, or ADE, of a second flaviviral infection [67,68]. Recent reports have demonstrated that dengue virus infection and viral cross-reactivity can result in antibody-dependent enhancement of a subsequent Zika virus infection [69]. In the development of potential Zika virus vaccines, ADE is being addressed by some investigators [70].

Recognizing the need for safe and efficacious Zika virus vaccines that can be used in pregnancy, The Ethics Working Group on ZIKV Research & Pregnancy, funded by the Wellcome Trust, was organized provided a series of recommendations to provide guidance in addressing the needs of pregnant women [71,72]. Their recommendations broadly outline three moral imperatives: (1) to develop a Zika virus vaccine that can be responsibly and effectively used during pregnancy, (2) to collect data specific to safety and immunogenicity in pregnancy for all Zika virus vaccine candidates to which pregnant women may be exposed, and (3) to ensure pregnant women have fair access to participate in Zika virus vaccine trials that offer a reasonably favorable ratio of research-related risks to potential benefits. In particular, the third imperative addresses the exclusion of pregnant and lactating women from past clinical trials and current usage of the Ebola vaccine during the West Africa and DR Congo epidemics. The report states [72], “*Denying pregnant women fair access to participate in ZIKV vaccine trials conducted in areas of active local transmission will unjustly exclude these women and their offspring from the prospect of direct benefit they may realize from receiving an investigational vaccine*”, and *“Fair access requires that eligibility to enroll or continue in a trial depend on reasonable assessments of the potential benefits of participation in relation to research-related risks for the woman and her future offspring. Fair access also requires that pregnant women are permitted to authorize or decline participation on their own*”.

In light of these recommendations, it was announced in February 2018 that a cooperative research effort by the University of Liverpool and University of Manchester, Public Health England and industry would develop and test new Zika vaccine candidates for safe use in pregnant women [73,74]—these two new vaccine candidates are based on a safe derivate of a pre-existing vaccine for smallpox. This research, financed by a £4.7 million award from Department of Health and Social Care and managed by Innovate UK, will progress to a Phase 1a first-in-human studies at the Royal Liverpool University Hospital’s Clinical Research Unit.

## 6. Are Pregnant Women Willing to Receive a Hypothetical Zika Virus Vaccine?

In Malaysia, there exists not only Zika virus transmission but also high levels of transmission of a related flavivirus infection, the dengue virus, both of which are transmitted by the same insect vector, *Aedes aegyptii*. In a cross-sectional study of pregnant women receiving antenatal care at the University Malaya Medical Center, the large majority (94%) expressed their willingness to receive a hypothetical Zika virus vaccine, perceiving that there were great benefits to receiving the vaccine [75]. Recommendation for receiving a Zika vaccine by a physician (odds ratio [OR] = 2.288; 95% confidence interval [CI] 1.093–4.793) and from friends or relatives (OR = 4.030; 95% CI 1.694–9.587), were both significant factors associated with a willingness to be vaccinated against Zika virus. A high level of desired participation by pregnant women for a vaccine is not unusual in countries where an infectious disease is endemic or potentially epidemic. 

In contrast, the United States has reported autochthonous transmission of Zika virus in only two states—Florida and Texas—and yet pregnant women there are still concerned about acquiring Zika virus infection when travelling to endemic areas. Recent studies in the United States have provided evidence that women are open and even accepting to receiving a hypothetical Zika virus vaccine during their pregnancy. In a 16-question survey of 100 pregnant women receiving prenatal care at a major university hospital in a non-endemic area for Zika virus transmission in the United States, Alholm et al. [76] found that a significant number of women would consider receiving a vaccine if one were available. Forty-eight percent (*n* = 48) of women surveyed expressed a strong sentiment to receiving a vaccine while they were pregnant, among whom 98% (*n* = 47) stated that a recommendation from their prenatal provider would be very important to them. Of the 52% (*n* = 52) of women who did not state a strong agreement with receiving a Zika vaccine while pregnant, 63% (*n* = 33) of them strongly agreed that a recommendation from their prenatal provider would be very important to them [76]. Interestingly, those women who voiced a strong sentiment to receiving a hypothetical Zika virus vaccine also had a positive attitude toward vaccination in general and among children. Those women having a strong acceptance of a Zika vaccine were also more likely to feel strongly about the importance of children being up to date on all their vaccinations (97% vs. 83%, *p* = 0.01) and the importance of getting recommended vaccinations during her pregnancy (97% vs. 79%, *p* = 0.003). In another study, from the United States of women’s attitudes towards hypothetical Zika virus vaccination during pregnancy, Goldfarb et al. [77] found that among a population of 129 pregnant women attending a prenatal clinic in Boston who volunteered to answer a questionnaire, 70% had expressed concern regarding Zika infection during pregnancy, with 44% having changed their travel planning as a result. Sixty-eight percent stated that they would be willing to consider participating in a clinical trial of an inactivated Zika vaccine; only 19% would agree to participate in a live virus vaccine trial (*p*-value < 0.0001)—a total of 77% of women would agree to participate in at least one hypothetical Zika vaccine clinical trial. Of all 129 pregnant women in this study, 51.6% would accept participating in a DNA vaccine clinical trial; 57% of women believed the inactivated vaccine during pregnancy was safe for their baby as compared with 26% for an attenuated live virus vaccine (*p*-value = 0.012). Those pregnant women who were willing trial participants were most motivated by a desire to protect their babies from Zika virus infection; fewer were strongly motivated by a desire to contribute to science. Most women who declined participation would reconsider given evidence of vaccine safety during pregnancy.

## 7. Conclusions

Zika vaccines should ultimately prove to be a critically important tool for the global prevention of Zika virus infection in pregnancy and the consequent infant morbidity and mortality. With the technical advancement of Zika vaccine candidates and as their clinical trials progress, it can only be hoped that pregnant women, their unborn fetuses and infants will be permitted to actively participate in these events. It cannot be determined whether vaccines are safe for use during pregnancy, and whether they exert a protective effect on the fetus in preventing congenital infection with its consequent morbidity and mortality, unless pregnant women are recruited and included in clinical testing and vaccine administration. When creating policy about these issues, it is important to critically evaluate vaccine safety in pregnancy in the context of the substantial risk of infection for the pregnant woman and her fetus in the absence of immunization. The denial of pregnant women to have fair access to voluntarily participate in vaccine trials occurring in regions where there is active local transmission of an infectious disease unjustly excludes these women, fetuses and infants from the potential direct benefit they may have from receiving an investigational vaccine.

## References

[1] Alvarado M.G., Schwartz D.A. (2017). Zika virus infection in pregnancy, microcephaly and maternal and fetal health—What we think, what we know, and what we think we know. Arch. Pathol. Lab. Med..

[2] Mead P.S., Hills S.L., Brooks J.T. (2018). Zika virus as a sexually transmitted pathogen. Curr. Opin. Infect. Dis..

[3] (2016). Zika and Guillain-Barré Syndrome. https://www.cdc.gov/zika/healtheffects/gbs-qa.html.

[4] Schwartz D.A. (2017). The origins and emergence of Zika virus, the newest TORCH infection: What’s old is new again. Arch. Pathol. Lab. Med..

[5] Schwartz D.A. (2017). Autopsy and postmortem studies are concordant. Pathology of Zika virus infection is neurotropic in fetuses and infants with microcephaly following transplacental transmission. Arch. Pathol. Lab. Med..

[6] Wheeler A.C. (2018). Development of infants with congenital Zika syndrome: What do we know and what can we expect?. Pediatrics.

[7] Moore C.A., Staples J.E., Dobyns W.B., Pessoa A., Ventura C.V., de Fonseca E.B., Ribeiro E.M., Ventura L.O., Neto N.N., Arena J.F. (2017). Characterizing the pattern of anomalies in congenital Zika syndrome for pediatric clinicians. JAMA Pediatr..

[8] Rice M.E., Galang R.R., Roth N.M., Ellington S.R., Moore C.A., Valencia-Prado M., Ellis E.M., Tufa A.J., Taulung L.A., Alfred J.M. (2018). Vital Signs: Zika-associated birth defects and neurodevelopmental abnormalities possibly associated with congenital Zika virus infection—U.S. territories and freely associated states. MMWR Morb. Mortal. Wkly. Rep..

[9] Satterfield-Nash A., Kotzky K., Allen J., Bertolli J., Moore C.A., Pereira I.O., Pessoa A., Melo F., Santelli A.C.F.E.S., Boyle C.A. (2017). Health and development at age 19–24 months of 19 children who were born with microcephaly and laboratory evidence of congenital Zika virus infection during the 2015 Zika virus outbreak—Brazil, 2017. MMWR Morb. Mortal. Wkly. Rep..

[10] Siedner M.J., Ryan E.T., Bogoch I.I. (2018). Gone or forgotten? The rise and fall of Zika virus. Lancet Public Health.

[11] Global immunization: Worldwide disease incidence. https://www.chop.edu/centers-programs/vaccine-education-center/global-immunization/diseases-and-vaccines-world-view.

[12] Faucette A.N., Pawlitz M.D., Pei B., Yao F., Chen K. (2015). Immunization of pregnant women: Future of early infant protection. Hum. Vaccin. Immunother..

[13] Bello O.O., Akinajo O.R., Odubamowo K.H., Oluwasola T.A. (2016). Lassa fever in pregnancy: Report of 2 cases seen at the University College Hospital, Ibadan. Case Rep. Obstet. Gynecol..

[14] Schwartz D.A., Anoko J.A., Abramowitz S. (2019). Pregnant in the Time of Ebola: Women and Their Children in the 2013–2015 West African Epidemic.

[15] Faucette A.N., Unger B.L., Gonik B., Chen K. (2015). Maternal vaccination: moving the science forward. Hum. Reprod. Update..

[16] Smithells R.W. (1978). Drugs, infections and congenital abnormalities. Arch. Dis. Child..

[17] Morelli S.S., Mandal M., Goldsmith L.T., Kashani B.N., Ponzio N.M. (2016). The maternal immune system during pregnancy and its influence on fetal development. Resp. Rep. Biol..

[18] The Thalidomide Tragedy: Lessons for Drug Safety and Regulation. https://helix.northwestern.edu/article/thalidomide-tragedy-lessons-drug-safety-and-regulation.

[19] Ortiz J., Englund J.A., Neuzil D.M. (2011). Influenza vaccine for pregnant women in resource constrained countries: a review of the evidence to inform policy decisions. Vaccine.

[20] Safety of Immunization During Pregnancy A Review of the Evidence. http://www.who.int/vaccine_safety/publications/safety_pregnancy_nov2014.pdf.

[21] Tsai T., Kyaw M.H., Novicki D., Nacci P., Rai S., Clemens R. (2010). Exposure to MF59-adjuvanted influenza vaccines during pregnancy—A retrospective analysis. Vaccine.

[22] Ouandaogo C.R., Yaméogo T.M., Diomandé F.V., Sawadogo C., Ouédraogo B., Ouédraogo-Traoré R., Pezzoli L., Djingarey M.H., Mbakuliyemo N., Zuber P.L. (2012). Adverse events following immunization during mass vaccination campaigns at first introduction of a meningococcal A conjugate vaccine in Burkina Faso, 2010. Vaccine.

[23] Zheteyeva Y.A., Moro P.L., Tepper N.K., Rasmussen S.A., Barash F.E., Revzina N.V., Kissin D., Lewis P.W., Yue X., Haber P. (2012). Adverse event reports after tetanus toxoid, reduced diphtheria toxoid, and acellular pertussis vaccines in pregnant women. Am. J. Obstet. Gynecol..

[24] Ryan M.A.K., Smith T.C., Sevick C.J., Honner W.K., Loach R.A., Moore C.A., Erickson J.D. (2008). Birth defects among infants born to women who received anthrax vaccine in pregnancy. Am. J. Epidemiol..

[25] Castillo-Solórzano C., Reef S.E., Morice A., Vascones N., Chevez A.E., Castalia-Soares R., Torres C., Vizzotti C., Ruiz Matus C. (2011). Rubella vaccination of unknowingly pregnant women during mass campaigns for rubella and congenital rubella syndrome elimination, the Americas 2001–2008. J. Infect. Dis..

[26] Yellow Fever. http://www.who.int/ith/vaccines/yf/en/.

[27] Merkatz R.B., Temple R., Subel S., Feiden K., Kessler D.A. (1993). Women in clinical trials of new drugs. A change in Food and Drug Administration policy. The Working Group on Women in Clinical Trials. N. Engl. J. Med..

[28] Evaluation of Gender Differences in Clinical Investigations—Information Sheet. https://www.fda.gov/RegulatoryInformation/Guidances/ucm126552.htm.

[29] Macklin R. (2010). Enrolling pregnant women in biomedical research. Lancet.

[30] Shields K.E., Lyerly A.D. (2013). Exclusion of pregnant women from industry-sponsored clinical trials. Obstet. Gynecol..

[31] Ethical Considerations in Biomedical HIV Prevention Trials. http://www.unaids.org/sites/default/files/media_asset/jc1399_ethical_considerations_en_0.pdf.

[32] Pregnant Women Should Be Included in Zika Virus Vaccine Research, New Guidance Says. https://healthtalk.unchealthcare.org/pregnant-women-should-be-included-in-zika-virus-vaccine-research-new-guidance-says/.

[33] Mor G., Cardenas I. (2010). The immune system in pregnancy: A unique complexity. Am. J. Reprod. Immunol..

[34] Strong A., Schwartz D.A., Schwartz D.A., Anoko J.A., Abramowitz S. (2019). Effects of the West African Ebola epidemic on health care of pregnant women—Stigmatization with and without infection. Pregnant in the Time of Ebola: Women and Their Children in the 2013–2015 West African Epidemic.

[35] Breman J.G., Heymann D.L., Lloyd G., McCormick J.B., Miatudila M., Murphy F.A., Muyembé-Tamfun J.J., Piot P., Ruppol J.F., Sureau P. (2016). Discovery and description of Ebola Zaire Virus in 1976 and relevance to the West African Epidemic during 2013–2016. J. Infect. Dis..

[36] (1978). Ebola haemorrhagic fever in Zaire, 1976. Bull. World Health Organ..

[37] Gomes M.F., de la Fuente-Núñez V., Saxena A., Kuesel A.C. (2017). Protected to death: systematic exclusion of pregnant women from Ebola virus disease trials. Reprod. Health,.

[38] Schwartz D.A., Schwartz D.A., Anoko J.A., Abramowitz S.A. (2019). Maternal and infant survival following Ebola infection—Their exclusion from treatment and vaccine trials and “Primum non nocere”. Pregnant in the Time of Ebola: Women and Their Children in the 2013–2015 West African Epidemic.

[39] Ebola Health Workers Face Life or Death Decision on Pregnant Women. https://www.health24.com/Medical/infectious-diseases/Ebola/Ebola-health-workers-face-life-or-death-decision-on-pregnant-women-20150115.

[40] Principles of Management for Pregnant Women with Ebola: A Western Context. www.rcog.org.uk/globalassets/documents/news/ebola-and-pregnancy-western.pdf.

[41] Alirol E., Kuesel A.C., Guraiib M.M., dela Fuente-Núñez V., Saxena A., Gomes M.F. (2017). Ethics review of studies during public health emergencies - the experience of the WHO ethics review committee during the Ebola virus disease epidemic. BMC Med. Ethics.

[42] Agua-Agum J., Ariyarajah A., Blake I.M., Cori A., Donnelly C.A., Dorigatti I., Dye C., Eckmanns T., Ferguson N.M., WHO Ebola Response Team (2015). Ebola virus disease among children in West Africa. N. Engl. J. Med..

[43] Ethical Issues Related to Study Design for Trials on Therapeutics for Ebola Virus disease. http://apps.who.int/iris/bitstream/handle/10665/137509/WHO_HIS_KER_GHE_14.2_eng.pdf;jsessionid=175A553938104AFE15DAB0A3C64A8839?sequence=1.

[44] Henao-Restrepo A.M., Longini I.M., Egger M., Dean N.E., Edmunds W.J., Camacho A., Carroll M.W., Dean N.E., Diatta I., Doumbia M. (2017). Efficacy and effectiveness of an rVSV-vectored vaccine in preventing Ebola virus disease: final results from the Guinea ring vaccination, open-label, cluster-randomised trial (Ebola Ça Suffit!). Lancet.

[45] DRCongo: Experts Call for Reversing the Decision to Deny the Ebola Vaccine to Pregnant Women. http://www.vaccineconfidence.org/drcongo-experts-call-for-reversing-the-decision-to-deny-the-ebola-vaccine-to-pregnant-women/.

[46] Caluwaerts S. (2017). Nubia’s mother: being pregnant in the time of experimental vaccines and therapeutics for Ebola. Reprod. Health..

[47] An Ebola Survivor Orphaned by Vaccine Policy. https://impactethics.ca/2016/11/25/nubia-an-ebola-survivor-orphaned-by-vaccine-policy/.

[48] Experts Call for Reversing the Decision to Deny the Ebola Vaccine to Pregnant Women. https://www.statnews.com/2018/08/27/experts-call-for-reversing-denial-of-ebola-vaccine-to-pregnant-women/.

[49] An ‘Indefensible’ Decision: Not Vaccinating Pregnant and Lactating Women in an Ebola Outbreak. https://www.statnews.com/2018/08/27/ebola-vaccine-pregnant-lactating-women/.

[50] Ebola Virus Disease Outbreak in Equateur Province, Democratic Republic of the Congo. https://ecdc.europa.eu/sites/portal/files/documents/17-05-2018-RRA-first-update-Ebola%20haemorrhagic%20fever-Democratic%20Republic%20of%20the%20Congo.pdf.

[51] In the Fight Against Ebola in the Congo, Pregnant Women Must Not be Forgotten. https://medicalxpress.com/news/2018-08-ebola-congo-pregnant-women-forgotten.html.

[52] (2018). New Ebola Outbreak Declared in North Kivu. https://www.msf.org/new-ebola-outbreak-declared-north-kivu.

[53] Ebola Virus Disease Democratic Republic of the Congo. http://apps.who.int/iris/bitstream/handle/10665/275658/SITREP_EVD_DRC_20181030-eng.pdf?ua=1.

[54] République Démocratique du Congo Situation épidémiologique dans les provinces du Nord-Kivu et de l’Ituri. https://mailchi.mp/sante.gouv.cd/ebola_kivu_1dec?e=f96cddba3b.

[55] WHO/UNICEF Zika Virus (ZIKV) Vaccine Target Product Profile (TPP): Vaccine to Protect Against Congenital Zika Syndrome for Use During an Emergency. http://www.who.int/immunization/research/development/WHO_UNICEF_Zikavac_TPP_Feb2017.pdf.

[56] Poland G.A., Kennedy R.B., Ovsyannikova I.G., Palacios R., Ho P.L., Kalil J. (2018). Development of vaccines against Zika virus. Lancet Infect. Dis..

[57] Barrett A.D.T. (2018). Current status of Zika vaccine development. Zika vaccines advance into clinical evaluation. Npj Vaccines.

[58] Ritter J.M., Martines R.B., Zaki S.R. (2017). Zika virus: Pathology from the pandemic. Arch. Pathol. Lab. Med..

[59] Rosenberg A.Z., Yu W., Hill A., Reyes C.A., Schwartz D.A. (2017). Placental pathology of Zika virus: Viral infection of the placenta induces villous stromal macrophage (Hofbauer cell) proliferation and hyperplasia. Arch. Pathol. Lab. Med..

[60] Schwartz D.A., Gajewski A., Petitt M., Fang-Hoover J., Padilla E., Llufrio L. Placental pathology of Zika virus infection from the Nicaraguan Zika Positives (NZP) Study: Preliminary findings. Proceedings of the 2nd International Conference of Zika Virus and Aedes Related Infections.

[61] Schwartz D.A. (2017). Viral infection, proliferation and hyperplasia of Hofbauer cells and absence of inflammation characterize the placental pathology of fetuses with congenital Zika virus infection. Arch. Gynecol. Obstet..

[62] Brazil’s Mothers Left to Raise Microcephaly Babies Alone. https://www.reuters.com/article/us-health-zika-women/brazils-mothers-left-to-raise-microcephaly-babies-alone-idUSKCN0WD21D.

[63] Schwartz D.A., Amarin Z. (2018). Pregnant and out of options—The quest for abortion in Latin America due to the Zika virus pandemic. Family Planning.

[64] Vaccines Protect Fetuses from Zika Infection, Mouse Study Shows. https://medicine.wustl.edu/news/vaccines-protect-fetuses-zika-infection-mouse-study-shows/.

[65] Sapparapu G., Fernandez E., Kose N., Cao B., Fox J.M., Bombardi R.G., Zhao H., Nelson C.A., Bryan A.L., Barnes T. (2016). Neutralizing human antibodies prevent Zika virus replication and fetal disease in mice. Nature.

[66] Magnani D.M., Rogers T.F., Beutler N., Ricciardi M.J., Bailey V.K., Gonzalez-Nieto L., Briney B., Sok D., Le K., Strubel A. (2017). Neutralizing human monoclonal antibodies prevent Zika virus infection in macaques. Sci. Transl. Med..

[67] Khandia R., Munjal A., Dhama K., Karthik K., Tiwari R., Malik Y.S., Singh R.K., Chaicumpa W. (2018). Modulation of dengue/Zika virus pathogenicity by antibody-dependent enhancement and strategies to protect against enhancement in Zika virus infection. Front Immunol..

[68] Martín-Acebes M.A., Saiz J.-C., de Oya N.J. (2018). Antibody-dependent enhancement and Zika: Real threat or phantom menace?. Front. Cell. Infect. Microbiol..

[69] Dejnirattisai W., Supasa P., Wongwiwat W., Rouvinski A., Barba-Spaeth G., Duangchinda T., Sakuntabhai A., Cao-Lormeau V.M., Malasit P., Rey F.A. (2016). Dengue virus sero-cross-reactivity drives antibody-dependent enhancement of infection with zika virus. Nat. Immunol..

[70] Dengue/Zika Vaccine that Avoids Antibody-Dependent Enhancement. https://gtr.ukri.org/projects?ref=972224.

[71] Zika Vaccines and Pregnant Women: Here’s What Ethics Experts Say. https://www.forbes.com/sites/brucelee/2017/07/10/zika-vaccines-and-pregnant-women-heres-what-ethics-experts-say/#592bb28b5ad6.

[72] Pregnant Women and the Zika Virus Vaccine Research Agenda: Ethics Guidance on Priorities, Inclusion, and Evidence Generation. http://guidance.zikapregnancyethics.org/wp-content/uploads/2017/08/Full+Guidance-Pregnant-Women-the-Zika-Virus-Vaccine-Research-Agenda_optimized.pdf.

[73] Zika Virus: Work Begins on Vaccine for Pregnant Women. https://www.bbc.com/news/uk-england-merseyside-43156952.

[74] New £4.7m Zika vaccine project launches. https://www.manchester.ac.uk/discover/news/new-47m-zika-vaccine-project-launches/.

[75] Wong L.P., Alias H., Hassan J., AbuBakar S. (2017). Attitudes towards Zika screening and vaccination acceptability among pregnant women in Malaysia. Vaccine.

[76] Alholm A., Ault K., Zwick R., Fitzgerald S., Satterwhite C. (2017). Pregnant women’s acceptance of hypothetical Zika vaccine. Open Forum Infect. Dis..

[77] Goldfarb I., Jaffe E., Lyerly A. (2017). Pregnant women’s attitudes toward Zika virus vaccine study participation. Obstet. Gynecol..

